# A Setup for Automatic Raman Measurements in High‐Throughput Experimentation

**DOI:** 10.1002/bit.70006

**Published:** 2025-07-11

**Authors:** Christoph Lange, Simon Seidel, Madeline Altmann, Daniel Stors, Annina Kemmer, Linda Cai, Stefan Born, Peter Neubauer, M. Nicolas Cruz Bournazou

**Affiliations:** ^1^ Chair of Bioprocess Engineering Technische Universität Berlin Berlin Germany; ^2^ Orientierungsstudium MINTgrün Technische Universität Berlin Berlin Germany

**Keywords:** automation, convolutional neural network, high‐throughput bioprocessing, Raman spectoscropy

## Abstract

High‐throughput (HT) experimentation is transforming biotechnology by enabling systematic exploration of complex multi‐dimensional experimental conditions. However, current analytical methods are often unable to handle the rapid pace of sample generation in HT workflows. This study presents an integrated system of physical devices and software to automate and accelerate Raman spectral measurements in HT‐facilities. The setup simultaneously handles eight parallel 50μL samples delivered by a pipetting robot, completing measurement, handling, cleaning, and concentration prediction within 45 s per sample. We introduce a machine learning model to predict metabolite concentrations from Raman spectra, achieving mean absolute errors of $0.27\;{\;\text{g L}\;}^{-1}$ for glucose and $0.06\;{\;\text{g L}\;}^{-1}$ for acetate during *Escherichia coli* cultivations. This approach enables consistent high‐throughput spectral data collection for fermentation monitoring, calibration, and offline analysis, supporting the generation of extensive datasets, enabling the training of more robust and generalizable machine learning models.

## Graphical abstract

- Record Raman spectra in High‐Throughput Bioprocess Development.
- Measure eight samples of 50μL fully automated.

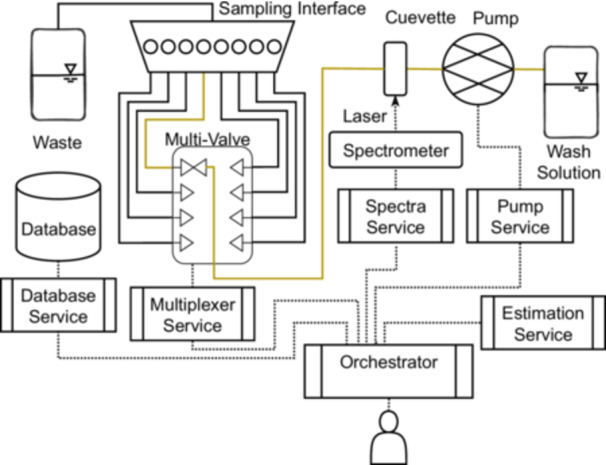

## 1 Introduction

High‐throughput (HT) cultivation systems have become essential for accelerating bioprocess development and biotechnological discovery. Automation, advanced feeding strategies, noninvasive sensors, and improved liquid‐handling technologies significantly enhance process reproducibility and monitoring capabilities (Hemmerich et al. 2018; Teworte et al. 2022; Qian et al. 2021; Anantanawat et al. 2019; Seidel et al. 2024). Over the past two decades, advances in robotics, automation, and molecular biology have expanded the scale and depth of HT experimentation in biotechnology (Janzen et al. 2019; Long et al. 2014; Kemmer et al. 2023).

However, a notable gap exists between the enormous capacity of HT‐cultivations and the lack of in‐depth analytical methods required for processes like untargeted phenotypic profiling (Franco‐Duarte et al. 2019). The prevailing analytical standard in HT‐experiments, relying primarily on targeted photometric assays, only provides limited insights into substrate and product concentrations (Wiendahl et al. 2008; Huber et al. 2009). Other parameters of utmost interest, for example, metabolic flux limitations or product quality, are not yet accessible in an integrated way.

To bridge this gap, Raman spectroscopy has emerged as a powerful Process Analytical Technology (PAT) to monitor substrates, metabolites, and product related parameters in a noninvasive manner (Lourenço et al. 2012; Wang et al. 2023; Nȩmcová et al. 2021; Allakhverdiev et al. 2022). To obtain concentrations from the Raman spectra, researchers usually use machine learning models. On larger scales, Partial Least Squares (PLS) works well for monitoring Chinese hamster ovary (CHO) cell fermentations (Esmonde‐White et al. 2022). Combined with Raman spectroscopy, PLS modeling is used to monitor metabolite concentrations in perfusion and cyclic processes (Schwarz et al. 2022; Voss et al. 2017). Moreover, Convolutional Neural Networks (CNN) predict fermentation concentrations from Raman spectra (Yan et al. 2020; Madsen et al. 2024; Khodabandehlou et al. 2024). Even though CNNs require larger datasets compared to PLS, CNNs are most often more accurate (Ibtehaz et al. 2023).

There already exists one system designed to measure Raman spectra in a high‐throughput context at the 15 mL (Rowland‐Jones et al. 2021) and 250 mL (Graf et al. 2022) scales. However, this system is limited to measuring only one sample at a time, with a recording duration of 1–5 min per spectrum. Moreover, proprietary hardware and software hinder modifications, integration into existing systems, and deployment of robust machine learning models like neural networks for predicting concentrations. Additionally, measuring Raman spectra in high optical density bacterial fermentations requires flexible modifications for sample preparation to enhance signal intensity (Rodriguez et al. 2023; Goldrick et al. 2020).

Considering the use case of multiple parallel mini‐bioreactors in an automated biolab, two main Raman monitoring strategies exist, each facing distinct challenges. The first strategy, involving external Raman probes positioned outside each reactor, often proves impractical due to limited optical access to centrally positioned reactors and low filling levels. The second strategy, employing external flow cells connected via fluidic interfaces, conflicts with utilizing single‐use reactors are small and typically lack the necessary integrated mounting ports.

To address these challenges, this study presents an automated Raman measurement system that is compatible with any HT‐cultivation platform equipped with a liquid‐handling robot, provided the robot is physically large enough to accommodate the measurement equipment. Initially, the hardware, software, and their integrated functionalities are described (Section 2). Subsequently, detailed measurement and calibration procedures aimed at improving measurement quality are presented (Sections 2.3 and 2.4). Furthermore, a machine learning model is proposed, and its utility is demonstrated through monitoring fermentations of *Escherichia coli* (Section 3). Finally, the developed system is compared to existing approaches, and its applicability for machine learning‐based process monitoring is discussed (Section 4).

## 2 Materials and Methods

An exhaustive exposition of the physical devices is initially presented in Section 2.1. Subsequently, we present more details about the software components in Section 2.2.

### 2.1 Devices

**Figure 1:**
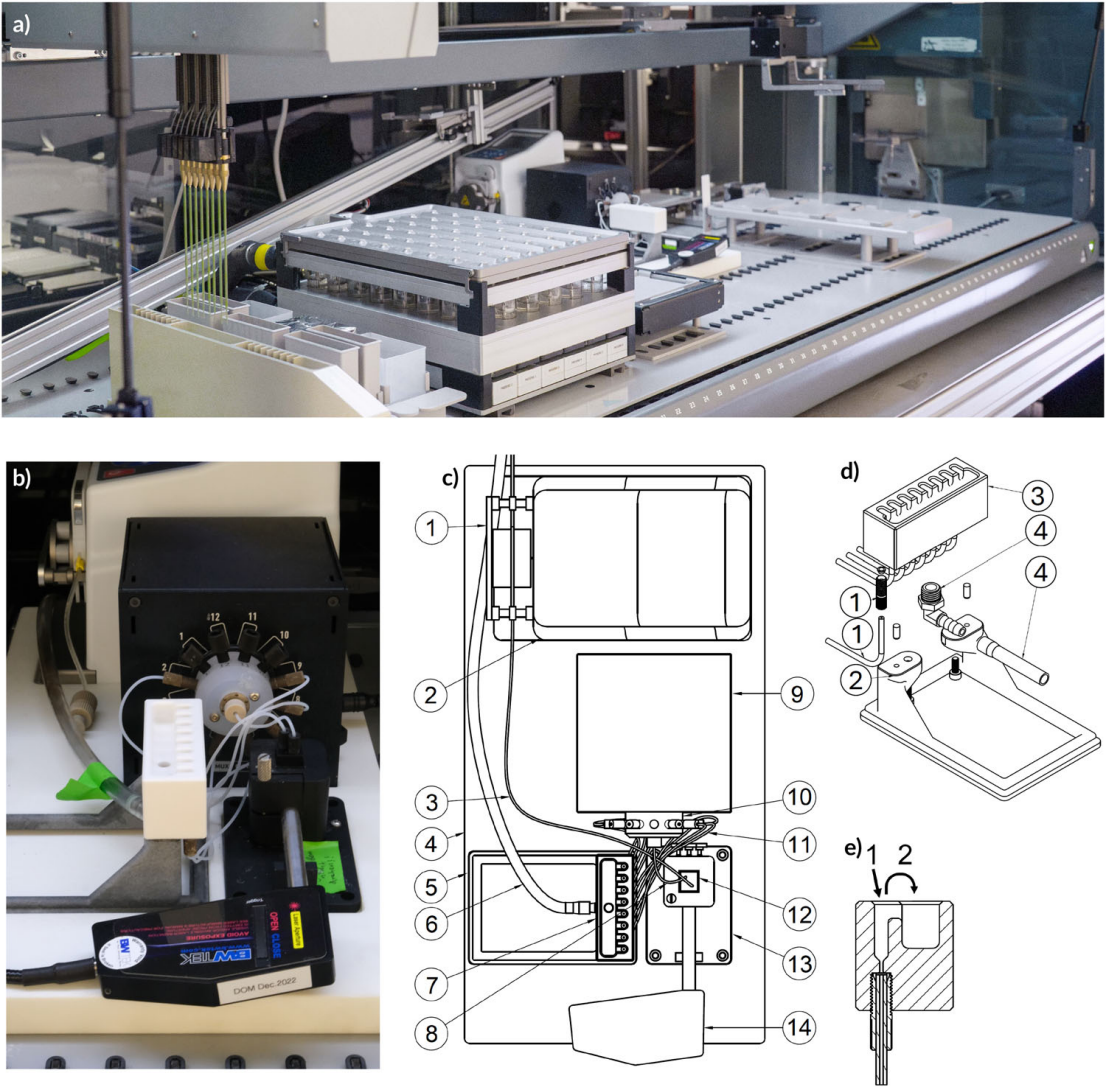
The integrated setup for automated Raman spectral measurements within a liquid handling robot. (a) shows the main liquid handling system, configured for automated HT‐fermentation based on a Tecan EVO 200 (Tecan Group, Männedorf, Switzerland) using a 48‐bioreactor platform (bioREACTOR 48; 2 mag AG, Munich, Germany; left hand side), with the developed integrated setup for automatic Raman measurements in HT‐experimentation (center). (b) gives a closer look onto the Raman measurement setup. It includes a PTFE pipetting interface for up to eight samples, connected to a flow‐through cuvette (Hellma GmbH & Co. KG, Müllheim, Germany) via microfluidic tubing and a multiplexer valve (Elveflow, Paris, France). Spectra recordings are conducted with a BAC102 probe (Metrohm AG, Herisau, Switzerland). (c) shows a schematic top‐down view of the system components: (1) peristaltic pump head, (2) pump case, (3) cleaning solution pumping tube, (4) device platform, (5) sampling interface rack, (6) wastewater tube, (7) sampling interface, (8) tube connecting valve and cuvette, (9) multiplexer valve case, (10) multiplexer head, (11) tubes connecting sampling wells and valve, (12) flow‐through cuvette, (13) BCR100A Raman cuvette holder (Metrohm AG, Herisau, Switzerland), and (14) fiber optic Raman probe assembly (FORPA). (d) provides a detailed drawing of the sampling interface. The interface includes eight sampling wells (3), each connected to the multiplexer valve via mounted tubes (1). An additional tube is connected to the overflow outlet (4), and the entire interface is mounted on a supporting rack (2). (e) shows a transverse cross‐sectional view of one sampling well of the pipetting interface. Its functions include (1) sample introduction via pipetting and (2) drainage of sample and cleaning solution via overflow, achieved by reverse pumping.

The system, integrated into an automated HT‐fermentation setup (Figure 1a) detailed in Figure 1b,c, consists of multiple units arranged within a liquid handling station to enable seamless transitions of samples. The sampling interface (Section 2.1.1) takes samples from the liquid handling robot (Section 2.1.6). Subsequently, each sample is transferred to the cuvette (Section 2.1.3) via a pump (Section 2.1.5).

#### 2.1.1 Interface

We designed a chemically inert sampling interface (Figure 1d) made from polytetrafluoroethylene (PTFE) to accept samples from a liquid handling robot. Each of its 8 wells can hold a volume of 125μL and the wells are spaced 9 mm apart, which is the same distance used for 96‐well microtiter plates. Each well is connected through a PTFE tube with an inner diameter of 0.508 mm to the multiplexer (Section 2.1.2) using microfluidic fittings. This setup allows the parallel acceptance of eight samples (arrow 1 in Figure 1e) that are stored in the wells until their sequential spectra measurement. The interface enables flushing samples into the waste‐water container via the overflow (arrow 2 in Figure 1e).

#### 2.1.2 Multiplexer Valve

We link the eight sampling interface wells (refer to Section 2.1.1) to the cuvette (see Section 2.1.3) by employing a 12‐to‐1 bidirectional valve multiplexer (Elveflow, Paris, France). Specifically, we utilize the multiplexer head's eight lower ports (Figure 1c, item 10) to minimize the distance between each port and the sampling interface. The multiplexer valve establishes a connection between the sampling interface wells (discussed in Section 2.1.1) and the cuvette (Section 2.1.3) through 65 mm PTFE tube. All tubes connecting the wells to the multiplexer ports measure 125 mm in length with a diameter of 0.508 mm. We optimized these tubes for minimal volumes and increased pump speed to enhance sample throughput.

#### 2.1.3 Cuvette and Cuvette Holder

**Figure 2:**
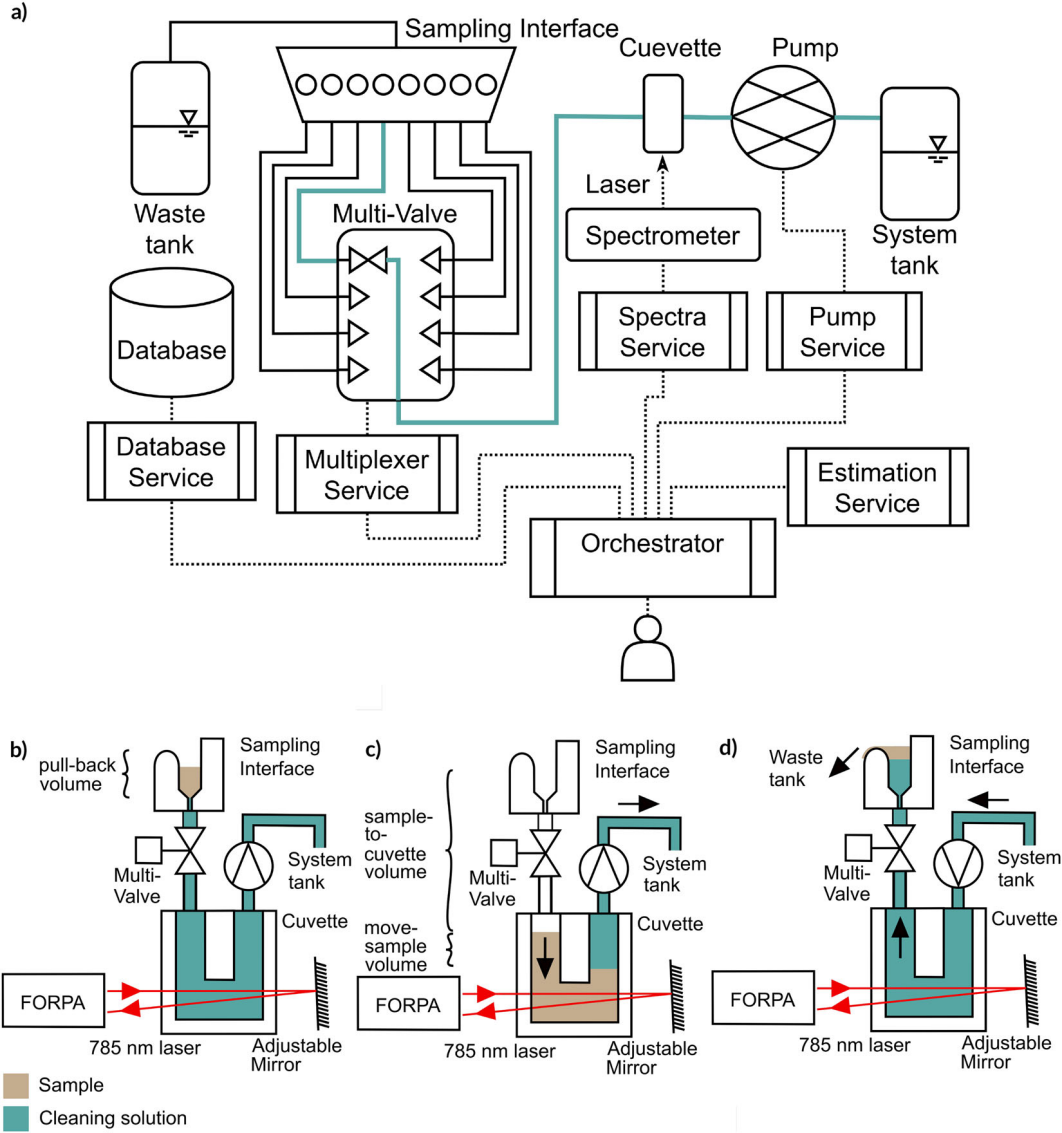
Liquid and Data Flow Between System Components: (a) shows the devices and software services required for automatic Raman measurements and their interactions. Solid black lines schematically represent physical connections (e.g., tubing), while dashed lines indicate device‐to‐device or software‐to‐device communication pathways. One of the possible liquid flows that is used during a measurement procedure is highlighted in blue and the stages within the measurement procedure are detailed in panels (b–d). Specifically, these stages are: (b) Sample positioned in the sampling interface awaiting measurement; (c) Active measurement within the cuvette; (d) Cleaning procedure for an individual channel. Each stage involves sample liquid, cleaning solution, the sampling interface (Figure 1c, item 7, Figure 1d, and Figure 1e), the Fiber Optic Raman Probe Assembly (FORPA, Figure 1c, item 14), multiplexer valve (Figure 1c, items 9 and 10), the cuvette with adjustable mirror (Figure 1c, item 12), the pump (Figure 1c items 1 and 2 (the symbol “>” indicates the direction of flow)), the system tank utilized as cleaning solution reservoir, and the waste tank.

The actual measurement of the Raman spectra occurs in a standard‐sized flow‐through cuvette (Figure 1c, item 10), positioned in the BCR100A Raman Cuvette Holder (Metrohm AG, Herisau, Switzerland; Figure 1c, item 13). The holder includes a mirror that reflects the laser beam back onto the sample (Figure 2b–d), enhancing the signal intensity and thereby reducing the spectrum recording duration. The cuvette, which serves as a flow cell, has a capacity of 18μμL and an optical path of 10 mm (Hellma GmbH & Co. KG, Article No. 1787128510‐40).

#### 2.1.4 Spectrometer

We use a Metrohm i‐Raman Plus 785 Spectrometer (Metrohm, Herisau, Switzerland) with an excitation wavelength of 785 nm, balancing signal strength and fluorescence (McCreery 2000). The spectra possess 2048 dimensions from $65\;{\;\text{cm}\;}^{-1}$ to $3350\;{\;\text{cm}\;}^{-1}$, with a laser power reaching 455 mW, facilitating quick recording. We use a BAC102 probe (Metrohm AG, Herisau, Switzerland; as shown in Figure 1c, item 14) with a flat quartz window and a working distance of 5.9 mm, which matches the dimensions of the cuvette holder (Section 2.1.3).

#### 2.1.5 Pump

For the purpose of transferring the sample from the sampling interface to the flow cell, a peristaltic pump was employed. Peristaltic pumps are recognized for delivering precise and reproducible pumping characteristics at high speeds in microfluidic systems (Tamadon et al. 2019). Furthermore, these type of pumps apply low shear forces to the fluid, thereby minimizing mixing between the measurement sample and the washing solution (Klespitz and Kovács 2014). In this specific process, a Masterflex Ismatec Peristaltic Pump, REGLO ICC (Avantor Inc., Radnor, Pennsylvania, USA), was utilized.

#### 2.1.6 Liquid Handling Robot

The system illustrated in Figure 1 is incorporated into a liquid handling station, namely a Tecan EVO 200 (Tecan Group, Männedorf, Switzerland), which facilitates automatic measurements during calibration, cultivation, or when utilized as an offline analyzer. This liquid handling robot is equipped with an arm featuring eight steel needles, enabling the transfer of samples into the eight wells of the sampling interface (Section 2.1.1). In addition, we use a minibioreactor system (bioREACTOR 48; 2mag AG, Munich, Germany) on the same liquid handler. Please note that any other minibioreactor system can be used as long as it can be operated by a liquid handling robot.

### 2.2 Software Components

Most of the devices discussed in Section 2.1, whose functionalities undergo changes throughout the measurement cycle, are designated with specific services, such as the spectrometer, pump, and multiplexer valve. These services enable remote management of these devices. In addition, we established two more services that interact with a relational database and predict substrate concentrations using a machine learning model. The coordination of the following components is handled by another service, the Orchestrator, as shown in Figure 2a:

- Spectrometer Service: Measures spectra
- Pump Service: Pumps back and forth
- Multiplexer Service: Switches valve position
- Database Service: Stores and loads data
- Estimation Service: Infers quantities from spectra

Each component is implemented as an independent microservice. The inter‐service communication occurs via gRPC, which provides fast communication and a language‐neutral interface definition for the Application Programming Interface (API). This modular architecture simplifies modifications to any component within the system configuration. The functionalities of each service are encapsulated within its own Python package. To enhance reusability, maintenance, and dependency management, each package is equipped with a Continuous Integration pipeline for testing the code, pre‐built deployment wheels, and comprehensive documentation. Further details of each package are presented in Table 1.

**Table 1:** Software Components: Here we specify the characteristics of all Python packages involved in a Raman measurement. We specify the release versions used, the fraction of code that is tested via unit tests, whether the packages use async concurrency, which operating system they can run on, if a Command Line Interface (CLI) is available and which kind of Application Programming Interface (API) is provided to reach a running server from the client side.

Components	Spectrometer Service	Pump Service	Multiplexer Service	Database Service	Estimation Service	Orchestrator
Release	0.1.9	0.1.6	0.2.3	0.5.3	0.1.5	0.4.8
Test coverage	93%	38%	44%	95%	87%	90%
Asynchronous	No	No	Yes	No	Yes	Yes
Operating System	Windows	Linux, Windows	Windows	Linux, Windows	Linux, Windows	Linux, Windows
CLI	No	Yes	Yes	Yes	No	Yes
API	Python	Python	Python	Python	Python	Python

Further information on the individual services is provided in the Appendix A. A detailed analysis of the Database Service and the Orchestrator is presented in the following sections.

#### 2.2.1 Database Service

The database interaction service offers a well‐defined interface to the relational database, which stores all critical biolab data, especially for HT‐experiments (Kaspersetz et al. 2022). The API includes endpoints for experiment management, spectra storage and export, sample creation and annotation, metadata addition, as well as persisting and loading machine learning models.

Utilizing this service offers several benefits relative to alternative services that directly access the database on the fly.

- Abstraction: The micro‐service encapsulates all complex logic inherent in the database scheme which eases its usage.
- Data Validation: The micro‐service validates input data before database entry.
- Consistency: Data is uniformly formatted across users.
- Security: Restrict direct database access to prevent data corruption.
- Maintainability: Changing Database logic requires updates only in one location.

More details of this service are available at https://bvt-htbd.gitlab-pages.tu-berlin.de/kiwi/tf3/raman-hive/.

#### 2.2.2 Orchestrator

The orchestrator operates on a server‐client model, with the server controlling all services described previously. The server side receives orders from the liquid handling station or users that trigger Raman measurements. The main features of the orchestrator include measuring Raman spectra and cleaning the entire setup to prepare it for a new measurement. This design abstracts complexity and simplifies the control of the individual services. More details about the orchestrator service are documented at https://bvt-htbd.gitlab-pages.tu-berlin.de/kiwi/tf3/raman-orchestrator/.

### 2.3 Measurement Procedure

Both physical elements and software components are connected in various ways for liquid and data transfer, as shown in Figure 2. To use all components for the measurement of Raman spectra of dimension D, we follow the steps depicted in Algorithm 1. This measurement process necessitates two distinct categories of variables: (1) parameters that remain invariant over extended periods to ensure measurement consistency; (2) input variables such as the number of spectra to be measured, which are subject to change between measurements. The following description outlines the processes that occur during the measurement procedure, as illustrated in Figure 2.

The measurement protocol is initiated through the orchestrator (Section 2.2.2) for the K sample (1≤K≤8), after pipetting these K samples into the interface, as shown in Figure 2b. Subsequently, the multiplexer (Section A.3) is adjusted to position k to connect the sample well to the cuvette. The pump (Section A.2) then moves the sample into the cuvette, as illustrated in Figure 2c. The subsequent phase of the process depends on the number of spectra N to be recorded for each sample. During spectra acquisition, the sample is moved by a volume of $V_{m}$, which we chose to be 20μL. This practice helps mitigate the presence of potential air bubbles in the spectrum and reduces heat transfer. Assuming a recording time of τ seconds per spectrum, we move the sample at a flow rate of $\frac{V_{m}}{N\tau }$, ensuring its transit until the end of recording the final spectrum. After each of the N spectra is recorded (Section A.1), they are stored in the database (Section 2.2.1) alongside the relevant metadata. Subsequently, all collected spectra are sent to the estimation service (Section A.4), and the orchestrator sends the predictions to the database service, such that the predictions are written to the database to allow other models to use them for adjusting feeding strategies (Krausch et al. 2022).

Finally, in the cleaning process (see Figure 2d), the initial action involves reversing the direction of the pump. During the pumping of the cleaning solution through the flow‐through cuvette, the samples are pushed back through the tubes into the overflow of the interface and drain into a waste tank. Upon completion of the cleaning phase, the solution is pumped forward once more by the pull‐back‐volume $V_{pb}$. This volume corresponds to the amount necessary to evacuate the well of the interface, as depicted in Figure 2b. This concluding procedure ensures that the well is empty and thus prepared to accommodate the subsequent sample.

Algorithm 1 Measuring *K* Samples: We describe the steps during the measurement of Raman spectra of *K* samples.

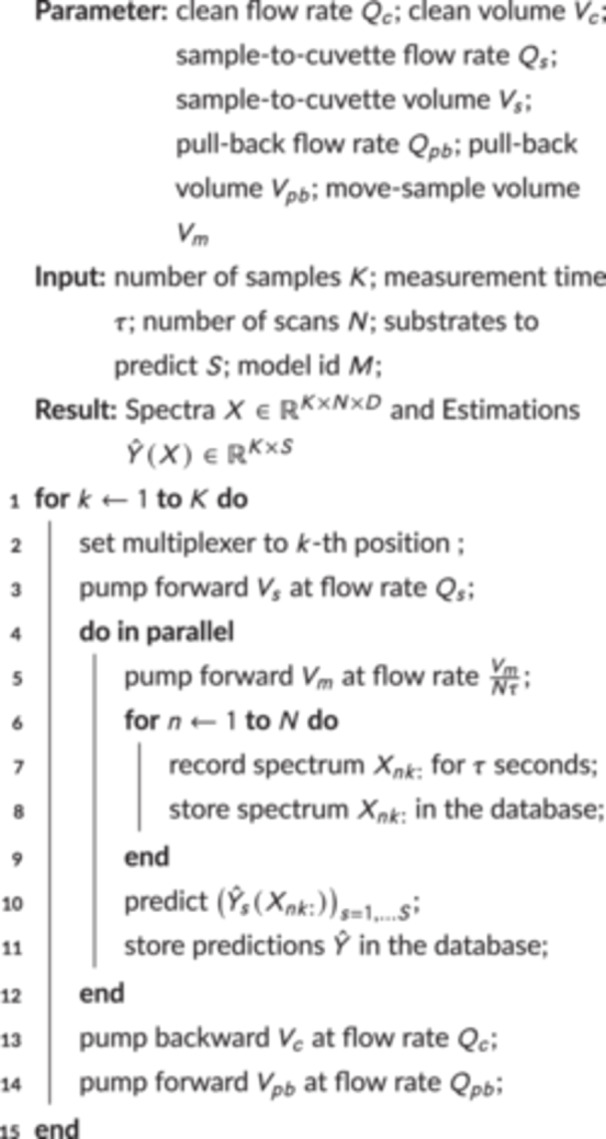


### 2.4 Calibration Procedure

According to Algorithm 1, several parameters affect the measurement process. For instance, proper positioning of the sample liquid within the cuvette is crucial to capture the correct sample spectrum instead of any remaining wash solution (refer to Figure 2c). Hence, we developed a calibration procedure that enhances the quality of the spectra obtained by the system concerning the parameters in the measurement process.

#### 2.4.1 Calibration Parameters

The measurement procedure (Algorithm 1) comprises seven adjustable parameters, each value can be selected from a broad interval of reasonable values. Since extensive testing all combinations simultaneously is infeasible, the sample‐to‐cuvette volume and pull‐back volume are prioritized, as these are crucial for proper liquid positioning in the cuvette (Figure 2c). We optimized the remaining five parameters individually. We set the flow rates $Q_{c}$, $Q_{s}$, and $Q_{pb}$ to their maximum reliable values and reduced them only in cases of spillover or reduced reproducibility.

We fixed the cleaning volume at 1250μL, based on control measurements conducted using water immediately after recording $50\;{\;\text{g L}\;}^{-1}\;{\;\text{MgSO}}_{4}$ solution. We observe no sulfate peaks at $980\;{\;\text{cm}\;}^{-1}$ anymore, which confirms effective cleaning.

A sample volume of 50μL was selected to fill the cuvette's internal volume (18μL) and to accommodate a move‐sample volume $V_{m}$ of 20μL to circulate the sample during measurement simultaneously, ensuring stable spectra and preventing local heating (Figure 2c).

#### 2.4.2 Calibration Metric

**Figure 3:**
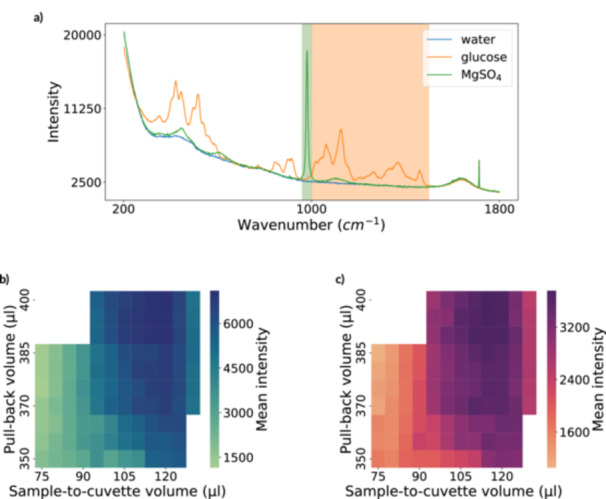
Raw spectra and corresponding heatmaps for calibration analysis. (a) Raw spectra of water, glucose, and magnesium sulfate highlighting key Raman peaks. (b) Mean intensity heatmap for magnesium sulfate (960–1000 ${\;\text{cm}\;}^{-1}$) across different sample‐to‐cuvette and pull‐back volumes. (c) Mean intensity heatmap for glucose (1000–1500 ${\;\text{cm}\;}^{-1}$) under the same conditions. The heatmaps show that for both substances, the best signal quality is achieved near 115μL to 120μL sample‐to‐cuvette volume and 395μL to 400μL pull‐back volume. For the selected sample‐to‐cuvette volume, the signal quality improves further when combined with higher pull‐back volumes.

To assess the impact of different parameter settings, we evaluated the mean intensity of Raman spectra from two different substances. We used $80\;{\;\text{g L}\;}^{-1}$ Glucose (D‐(+)‐glucose monohydrate; Carl Roth, Karlsruhe, Germany) and $50\;{\;\text{g L}\;}^{-1}\;{\;\text{MgSO}}_{4}$ (Magnesium Sulfate Heptahydrate; Carl Roth, Karlsruhe, Germany), which we chose for their biotechnological relevance and high Raman activity. The glucose intensity averaged over the wavenumber range $1000\;{\;\text{cm}\;}^{-1}$ to $1500\;{\;\text{cm}\;}^{-1}$, and ${\;\text{MgSO}}_{4}$ averaged over $960\;{\;\text{cm}\;}^{-1}$ to $1000\;{\;\text{cm}\;}^{-1}$ (see Figure 3b).

Taking the average intensity helps account for variations such as sample placement discrepancies or the formation of air bubbles, which may happen when the pull‐back volume is excessive, leading to an air gap between the sample and the wash solution (Figure 2b). Consequently, we avoid using ratiometric criteria like the signal‐to‐noise ratio, as these do not effectively account for inter‐sample variations.

#### 2.4.3 Measurement Procedure

We performed a grid search varying the sample‐to‐cuvette volume from 75μL to 140μL and the pull‐back volume from 350μL to 400μL, both in 5μL steps. Since both volumes are interdependent, their optimal values must be determined jointly to ensure reproducible and consistent spectral quality.

For each set of parameters, spectra were measured over three replicate rounds per well. Specifically, we initially completed the first measurement round for all parameter sets, followed by the second round, to reduce the impact of temporal distribution changes. To maintain consistent laser power, the laser remained continuously on throughout the entire calibration process.

### 2.5 Maintenance

To uphold the quality of the measurements, regular maintenance is imperative. Of particular importance is the cleaning of the system, which involves flushing the setup with water and eliminating residues using anti‐static cotton swabs saturated with isopropanol. The integrity of the spectral data can be verified by analyzing samples of demineralized water and comparing the configuration and intensity of the obtained spectra with a high‐quality reference spectrum. When working with cells, the system should be flushed afterwards with a 5% hydrochloric acid (AnalaR NORMAPUR, VWR Chemicals, Darmstadt, Germany) solution to eliminate mineral deposits, metal oxides, and other contaminants. Subsequently, we performed repeated cleaning of the apparatus with demineralized water.

### 2.6 Machine Learning Model

Since the pure spectra provide limited utility for monitoring fermentations, we developed a machine learning model to predict concentrations from the spectra. To validate the effectiveness of this system, we conducted tests using the setup alongside a machine learning model in an actual fermentation process.

#### 2.6.1 Fermentation Setup

We used the Raman measurement setup described in this study in a fermentation process utilizing *E. coli* which included a batch phase lasting approximately 4 h and a glucose‐limited feeding phase, also lasting around 4 h. We conducted the experiment according to the procedure specified in (Krausch et al. 2022). Sample collection occurred approximately every hour using the liquid handler that hosts both the mini‐bioreactors as well as the Raman setup (Figure 1a). These samples underwent manual centrifugation (ROTANTA 460 Robotic, Hettich, Tuttlingen, Germany) at $4^{{}^{\circ}}$C and 2000 rpm for 4 min to separate the cellular matter from the supernatant. We recorded that the manual process of transferring the plate with samples to the centrifuge, extracting the supernatant, and placing it within reach of the liquid handler took roughly 110 s.

The separation of cells and supernatant is carried out for two main reasons. Firstly, the supernatant undergoes analysis to measure concentrations of glucose and acetate using a Cedex Bio HT‐Analyzer (Roche Diagnostics Deutschland GmbH, Mannheim, Germany) equipped with GLC3B and AC2B kits for glucose and acetate. Secondly, the robot directs the supernatant to the sampling interface, where we record two Raman spectra with a duration of 10 s each. The cell removal notably improves the signal strength of the metabolites.

#### 2.6.2 Model Architecture

For infering concentrations from spectra we use a CNN, well‐regarded for identifying intricate patterns in fields such as computer vision (Bishop and Bishop 2024) and spectral analysis. For spectra we implement 1‐D convolutions to construct filters targeting adjacent points within a single spatial dimension, unlike the filter applications in 2‐D or 3‐D for images and videos. We use the same CNN structure as (Lange et al. 2025), consisting of 8 blocks to reduce spatial dimensions. This model does not require any classic preprocessing steps, as the model learns an appropriate encoding within the convolutional blocks. This strategy integrates cutting‐edge techniques, including residual connections (He et al. 2015) enhanced with a scaling factor (Bachlechner et al. 2020), depthwise separable convolutions (Chollet 2017), and exponential linear units as activation functions (Clevert et al. 2016).

#### 2.6.3 Model Training

During training, the network parameters are iteratively updated to capture the peak patterns of the substances in the sample more effectively. To enhance the robustness of our neural networks, we use data augmentation techniques that increase their generalization capabilities during model training. In particular, we use MixUp (Zhang et al. 2018) and implemented further augmentations of the spectra, such as baseline shifts and modifications to the slope of the spectra (Lange et al. 2025).

For parameter updates, we apply the Adam optimizer with decoupled weight decay (Loshchilov and Hutter 2019). We utilize a learning rate schedule that includes a warm‐up period of 20 epochs, followed by an exponential decay (Li and Arora 2019).

#### 2.6.4 Evaluation Procedure

To assess the CNN with respect to the 24 fermentation trials, we divided the experiments into three groups at random: a training set consisting of 8 experiments, a validation set with another 8 experiments, and a testing set containing the remaining eight experiments. Given that neural networks require substantial training data, we incorporated a publicly available data set (Lange et al. 2025), which comprises 2261 annotated spectra with glucose and acetate labels, into the training set. These spectra were derived from 8 different spectrometers, so we harmonized them in two ways. First, we interpolated the spectra to ensure they had consistent dimensions. Second, we applied Standard Normal Variate normalization to ensure their values were in a comparable range.

Our approach involved utilizing the division of fermentation experiments alongside supplementary training data to execute hyperparameter tuning for our CNN. We trained the model using each hyperparameter setup on the training data set and assessed its performance on a validation data set. To maintain the cost of hyperparameter tuning at a reasonable level, we employed Bayesian optimization and Hyperband (Falkner et al. 2018) with a reduction factor of 3. Upon concluding hyperparameter tuning, the optimal hyperparameter configuration was evaluated using the test data set. This methodology ensures an impartial estimation of prediction error when applied to unseen spectra.

## 3 Results

We show the best measurement conditions that we obtained from our calibration procedure (Section 3.1), get the durations of the individual components (Section 3.2), and show how we used them to measure spectra during a fermentation of *E. coli* including predictions from our machine learning model (Section 3.3).

### 3.1 Calibration Results

This section examines the results of the calibration method that aims to provide reliable high‐quality spectra from the analytes. According to the calibration procedure explained in Section 2.4 we depict the signal intensity in Figure 3 under various conditions. These measurements are conducted for both glucose (Figure 3c) and magnesium sulfate (Figure 3b) to determine if the findings apply to various substances.

For both substances, the spectral intensity increases with greater pullback volumes. This trend occurs because larger pullback volumes decrease the amount of wash solution remaining in the wells after the washing steps, resulting in fewer dilutions of the analyte. For the sample‐to‐cuvette volume, the right amount ensures that the sample is placed directly in front of the laser (Figure 2c).

We observe a high signal intensity for the sample‐to‐cuvette volume to be 105μL to 120μL. Volumes below this range have a lower intensity, probably resulting from an insufficient amount of sample reaching the cuvette. On the other hand, volumes above 120μL exhibit a steep decline in signal intensity, probably caused by the increase in the fraction of the sample that is pumped beyond the cuvette.

For glucose in Figure 3c, the highest signal intensity occurs at 115μL sample‐to‐cuvette and 395μL pull‐back volume. In contrast, Figure 3b shows that magnesium sulfate provides the best calibration at a pull‐back volume of 400μL and a sample‐to‐cuvette volume of 120μL. However, the setting with a 115μL sample‐to‐cuvette volume and a 395μL pull‐back volume offers the third largest intensity. As a result, we opted for this setting to align with the glucose calibration.

**Figure B1:**
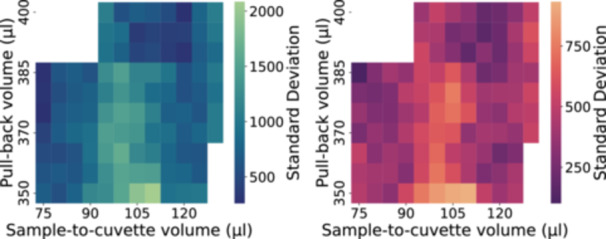
*Standard deviation of Raman calibration* For magnesium sulfate (left) and glucose (right) we measured at least 40 samples using different settings for the pull‐back volume and the sample‐to‐cuvette volume. Here we show the standard deviation of the signal.

Furthermore, the standard deviations of the signal intensities (Figure B1) are examined to ensure that observations with high averages are not due to outliers exhibiting extremely high intensity. For the setting of 115μL sample‐to‐cuvette volume and a 395μL pull‐back volume, we trust the high signal intensity as we observe a generally low standard deviation.

### 3.2 Measurement Duration

In this section, we outline the time required for each step of the Raman measurement process that we described in Algorithm 1 in more detail.

- Duration switching valve position: 1.3 s
- Duration pumping sample to cuvette: 3.6 s
- Duration cleaning channel: 10 s
- Duration pull back: 4.74 s
- Time spent in software including prediction: 0.56 s
- Total overhead time: 20.2 s
- Measuring a Raman spectrum: 10 s

As all the steps except the measurement duration per spectrum have a fixed duration, there is always an overhead around 20.2 s that comes with each sample. The exposure time of each Raman spectrum and the number of spectra to be measured are free to choose for the user. In our experiments, we used 10 s per spectrum and recorded two spectra per sample, but one can increase both numbers to improve the signal‐to‐noise ratio.

### 3.3 Fermentation Results

We measured Raman spectra in 24 *E. coli* fermentations. Here we look at the spectra of the fermentation and the results of the CNN on the test data set.

**Figure 4:**
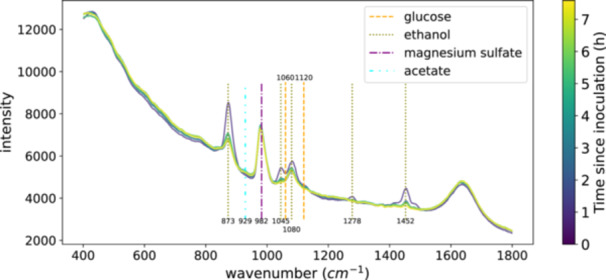
Raman spectra during *E*. *coli* fermentation. The spectra show the supernatant from one *E. coli* fermentation measured with the setup described in this paper. Numbers above or below the vertical lines indicate peak positions obtained from the literature (Emin et al. 2020; Jiang et al. 2020; Frost and Kloprogge 2000; Mathlouthi and Vinh Luu 1980; Wang et al. 2006).

Looking at the spectra in Figure 4 we observe a stable baseline of the spectra, along with temporal variations in peak intensities. In particular, several distinguished peaks can be linked to ethanol (Emin et al. 2020), which serves as a disinfectant for the needles of the liquid handling station. Over time, the intensity of these ethanol peaks decreases, corresponding to an increase in the cell count that consumes some of the ethanol during the sampling procedure. Our observations of the acetate peak (Frost and Kloprogge 2000) indicate a slight increase in acetate, an aerobic overflow metabolite and anaerobic by‐product, over the course of the fermentation. Glucose, being the primary carbon source, diminishes over time. Magnesium sulfate, which acts as a cofactor to enhance microbial substrate‐to‐product conversion (Gotsmy and Strobl 2023), shows a slight decrease.

**Figure 5:**
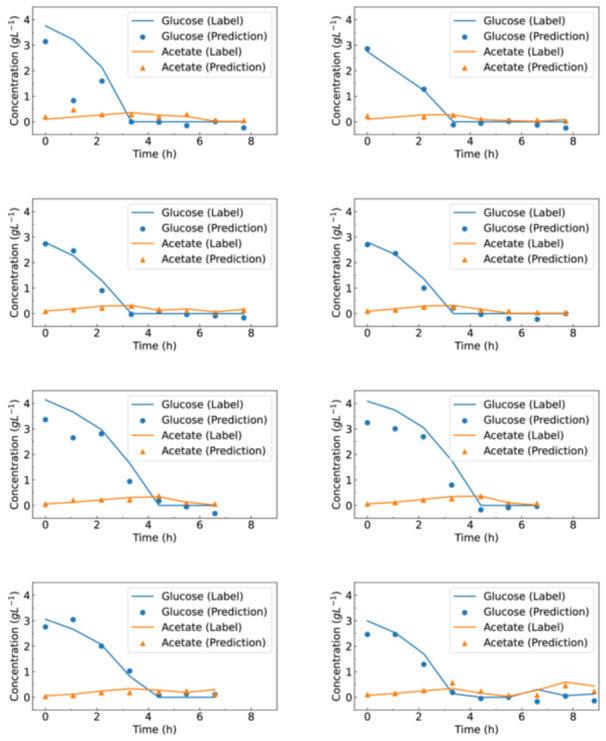
*E. coli* fermentation trajectories. Time series data of glucose and acetate concentrations from the eight experiments in the test data set. Solid lines indicate concentrations measured via the HT‐Analyser, while symbols represent machine learning predictions for the same concentrations.

To evaluate the performance of the machine learning model, we compared the predictions against the measurements of the HT‐Analyser. In particular, Figure 5 depicts both the measurements and the CNN predictions for all 8 cultivations of the test data set. Looking at the residues, the predictions of acetate are more accurate than those for glucose. This is confirmed by the mean absolute error of $0.27\;{\;\text{g L}\;}^{-1}$ for glucose and $0.06\;{\;\text{g L}\;}^{-1}$ for the acetate predictions.

## 4 Discussion

In this study, we developed and tested an automated procedure for measuring Raman spectra at‐line with a liquid handling robot during HT‐fermentations, calibration procedures, or offline analytics.

### 4.1 Comparison to Existing Systems

Our Raman spectroscopy system offers advantages over existing commercial equipment. By utilizing a multi‐well design in combination with an 8‐needle‐based liquid‐handling robot and our in‐house software, we achieve significant improvements in both throughput and flexibility.

A major strength of the presented system is its ability to sample eight wells simultaneously and measure the samples in quick succession, significantly reducing the overall measurement time per sample compared to currently available Raman devices for systems such as the Ambr 15 and Ambr 250 (Sartorius, Germany), as the system avoids the idle time when taking a new sample. Furthermore, using the interface as a short‐term storage for the samples minimizes the time share of the liquid handling robot that is used for Raman measurements. This is particularly important in faster‐paced processes, such as *E. coli* fermentations, where frequent sampling is needed alongside other tasks like feeding and pH control.

Our self‐developed hardware and software design allows for customization and integration of the device into existing laboratory environments. Unlike commercial systems that rely on proprietary platforms, the system is open source and therefore readily adaptable for everybody. For instance, we implement advanced data analysis tools, such as neural networks, to improve predictive accuracy and extract deeper insights from the collected spectra. Or we could simply add a second spectrometer to the measurement flow. This flexibility supports a broader range of applications and enhances the system's utility beyond standard Raman measurements.

In summary, the combination of parallel sampling, efficient workflow management, and customization makes the Raman spectroscopy system a highly effective and versatile tool, particularly for parallel high‐throughput and time‐sensitive analytical tasks.

### 4.2 Benefits for Machine Learning Models

We trained a CNN model that achieved a similar error rate for glucose as reported in (Rowland‐Jones et al. 2021), but demonstrated even greater accuracy when assessing acetate. While these results are satisfactory for fermentation monitoring, the training setup of the model is not ideal concerning the data ratio. That is, when training the CNN we used a lot more spectra from different contexts compared with the actual fermentation experiments. Even though the additional spectra regularize the model, adding more annotated Raman spectra recorded under the same conditions will allow for the development of more sophisticated and accurate models in the future (Arend et al. 2020). For example, (Passos and Mishra 2023) shows that the dry matter content of various fruits can be predicted more accurately using increasing numbers of near‐infrared spectra, and we anticipate this phenomenon to occur for Raman spectra as well.

Here, the fully automated setup presented in this study will be of great use to generate more data. Currently, complex models such as neural networks primarily address classification problems (Luo et al. 2022) in the domains of medicine, biology, and biotechnology (Sohn et al. 2020; Wu et al. 2021; Hu et al. 2022; Dong et al. 2019; Maruthamuthu et al. 2020) that require fewer data compared to regression problems. With bigger datasets being available, we will be able to combine them to train more sophisticated models. For example, we could use transformer architectures that are more common in image or spectral data (Vaswani et al. 2023; Chang et al. 2024; Koyun et al. 2024; He et al. 2021).

Additionally, uniform measurement conditions in the cuvette (Section 2.1.3) increase consistancy. As demonstrated here, the system can be used on the 15 mL scale, but one could also use it on a scale of 150 mL like (Kaspersetz et al. 2022). That promotes reusing data, thereby reducing the necessity for a large amount of calibration data in new scenarios.

### 4.3 Design Considerations

With regard to the design of the measurement setup, there are alternative approaches that we could have followed. Therefore, we explain our thought process that lead to the current approach.

At first, we have chosen to keep the tubes filled with liquid. This decision is grounded in the fact that the pump achieves more consistent results when pumping liquid rather than a combination of samples and air, particularly when the majority of the tube is occupied by air. Consequently, the placement of the sample in the cuvette is more consistent with the presence of liquid in the tubes, thereby resulting in more reliable spectra.

Second, with respect to the order of steps within the measurement procedure, we considered various alternatives. For instance, it is not imperative to perform the cleaning of each channel immediately following the measurement of the sample. An alternative approach would be to measure the samples across all channels before conducting the cleaning for each channel individually in a stop flow manner. That option would decrease the lag time between the samples placed in the interface and the Raman spectra of the last sample being measured. Nevertheless, there are two significant drawbacks to this approach. On the one hand, the cleaning of the cuvette and the tubes between the cuvette and the multiplexer valve is constrained, as it relies solely on the washing solution already present in the tubes of the succeeding channel between the interface and the cuvette. Therefore, depending on the concentrations of substances present in the samples the amount of washing solution may not suffice. On the other hand, this strategy necessitates altering the position of the multiplexer 2K times per measurement cycle, in contrast to K times required in the current arrangement, thereby resulting in a saving of 1.3 s per channel, which is the average time the multiplexer valve needs to change its position.

Third, one of our concerns was that residual cleaning solution in the tubing dilutes the sample and thereby affect the Raman spectra. However, the tubing used has an inner diameter of only 0.508 mm, minimizing the volume where carry‐over could occur. In practice, such dilution is not problematic as long as it is consistent. The low standard deviation in the calibration procedure and the high accuracy of the machine learning model suggest that any dilution effects are systematic and reproducible.

### 4.4 Outlook

We developed the setup for the usage within automated fermentations for high‐throughput bioprocess development of up to 48 mini bioreactors in parallel (Kemmer et al. 2023). Therefore, everything is optimized regarding automation, reliability and speed. However, we designed the system very modular so that we can use it in different applications.

For example one can use the system for monitoring enzymatic reactions within biocatalysis or organic chemistry that are conducted on a liquid handling robot. As Raman spectroscopy can provide real‐time, label‐free detection of molecular changes by analyzing vibrational fingerprints of substrates, intermediates and products, and can thus greatly help in understanding how enzymes act on the molecular basis (Carey and Pusztai‐Carey 2021). Moreover, Raman spectroscopy can track redox changes in coenzymes such as NADH/NAD during dehydrogenase‐catalyzed reactions (Chen et al. 1987) or detect structural modifications in peptides (Sahoo et al. 2016) and polysaccharides (He et al. 2020) during enzymatic hydrolysis. It can also be used to analyze the concentrations of reaction components.

In all these applications, our Raman system, integrated into the liquid handling station, facilitates diverse pretreatment methods for samples. For instance, one could dilute a sample to a specific optical density for characterizing inclusion bodies, homogenize samples to analyze intracellular contents or add nano particles for Surface Enhanced Raman Spectroscopy (Lin et al. 2021).

## Author Contributions

**Christoph Lange:** conceptualization, data curation, formal analysis, investigation, methodology, software, visualization and writing – original draft. **Simon Seidel:** conceptualization, methodology, resources, visualization and writing – original draft. **Madeline Altmann:** conceptualization, data curation, formal analysis, investigation, methodology, visualization and writing – original draft. **Daniel Stors:** conceptualization, data curation, formal analysis, investigation, methodology, visualization and writing – original draft. **Annina Kemmer:** investigation, resources, supervision and writing – review and editing. **Linda Cai:** investigation, resources, supervision and writing – review and editing. **Stefan Born:** conceptualization, supervision and writing – review and editing. **Peter Neubauer:** supervision, funding acquisition and writing – review and editing; **M. Nicolas Cruz Bournazou:** supervision, funding acquisition and writing – review and editing.

## Conflicts of Interest

The authors declare no conflicts of interest.

## Data Availability

The data that support the findings of this study are openly available in Raman Kiwi Experiments at https://git.tu-berlin.de/bvt-htbd/kiwi/tf3/raman-kiwi-experiments.

## Appendix A Additional Software Components

### A.1 Spectrometer Service

The spectrometer service is specifically tailored for the Metrohm i‐Raman Plus spectrometer as described in Section 2.1.4. It offers an API that allows measurements of spectra by specifying either the laser intensity or the measurement duration. More information on this service can be found at https://bvt-htbd.gitlab-pages.tu-berlin.de/kiwi/tf3/raman-spectrometer-server/.

### A.2 Pump Service

The pump service facilitates communication with the specified Ismatec pump (Section 2.1.5) with which communication occurs through a serial connection. This service provides an API to the pump, allowing the configuration of parameters such as flow rate, volume, direction, delay, and the option to wait for pump completion. In addition, it offers an interface to modify the default settings and perform a health check. Additional details on this service can be found in https://bvt-htbd.gitlab-pages.tu-berlin.de/kiwi/tf3/ismatec-pump/.

### A.3 Multiplexer Service

The multiplexer valve service facilitates altering the valve's position. It is tailored for the Elveflow valve, which is elaborated upon in Section 2.1.2. The API for adjusting the valve includes a feature to set the direction, as well as a health check functionality. Additional information about this service can be found at https://bvt-htbd.gitlab-pages.tu-berlin.de/kiwi/tf3/multiplexer-valve/.

### A.4 Estimation Service

The estimation service infers substance concentrations from a Raman spectrum. At startup, it loads specified models from the database via the API in Section 2.2.1, performs preprocessing, and allows predictions for all trained parameters. More details are available at https://bvt-htbd.gitlab-pages.tu-berlin.de/kiwi/tf3/raman-estimate/.

## Appendix B Standard Deviation of the Calibration Results

Here we provide the standard deviation of the signal intensities during the calibration runs. In Figure B1 we show the results for each setting for glucose and magnesium sulfate.

## Appendix C Standard Operating Procedure (SOP)

This is an SOP for the use of the Raman measurement setup during a cultivation in the 2mag system. It gives instructions on how to prepare the system for an experiment and how to handle it during an experiment. It also gives short maintenance instructions and a short troubleshooting help.

### C.1 Raman Paramters

Table C1 gives an overview of the experiment and how the different variables of the system have been set. The standard values for most of the variables are given and should not need to be changed for a standard experiment.

**Table C1:** Raman parameters (standard values given).

Parameter	Value	Details/Comments
Model (device id)		Refer to SQL data base to find device‐id of the model that estimates concentrations from spectra
run‐id		Refer to SQL data base to select your created run or create new run with raman‐hive commands (see documentation)
Substances of interest	Glucose, Acetate, Phosphate, Glycerol, Nitrate, Magnesium Sulfate	The substances that the model will predict the concentration of
Measurement method	manual/automatic	Manual: samples need to be pipetted into the pipetting interface by hand; automatic: samples are pipetted by the liquid handling robot
Sample‐to‐cuvette‐pump‐volume [L]	1.15e‐4	Volume pumped to bring the sample from the interface to the cuvette for measuring
Pull‐back‐pump‐volume [L]	3.95e‐4	Volume to empty the well after flushing
Clean‐pump‐volume [L]	1.25e‐3	Volume pumped for cleaning after a sample has been measured
Sample‐to‐cuvette‐flow‐rate [${\;\text{L min}\;}^{-1}$]	7.5e‐3	Flow‐rate corresponding to sample‐to‐cuvette‐pump‐volume
Pull‐back‐flow‐rate [${\;\text{L min}\;}^{-1}$]	5e‐3	Flow‐rate corresponding to pull‐back‐pump‐volume
Clean‐flow‐rate [${\;\text{L min}\;}^{-1}$]	7.5e‐3	Flow‐rate corresponding to clean‐pump‐volume
Move‐sample‐volume [L]	2e‐5	Volume the sample is pumped forwards during measurement
Duration [s]	10	Duration of one scan
Num_scans	2	Number of scans performed per measurement
Sample‐volume [μL]	50	Volume of sample pipetted into the interface
Number‐of‐wells	8	Number of wells used for the measurement
Time and date of water check		The time and date at which the water check has been performed
Run‐id of the water check		The run‐id used to store the spectra for the water check before the cultivation

### C.2 Pre‐Experiment Preparation

In Table C2 we present in more detail the preparations that need to be done a few days before the experiment.

**Table C2:** Pre‐experiment preparation.

Step	Details
Train model	Training a model and save it into the database Check out the notebook to train a model: https://git.tu-berlin.de/bvt-htbd/kiwi/tf3/raman_master/-/blob/main/notebooks/neural_networks/train_nn_that_predicts_std_mock_dataset.ipynb?ref_type=heads Store model in database https://bvt-htbd.gitlab-pages.tu-berlin.de/kiwi/tf3/raman-hive/cli.html#raman-hive-client-store-model Remember the device‐id of your model
Incorporate Raman script into your Tecan script	We wrote a script that reads the appropriate information from a configuration file and creates a command for conducting Raman measurements via EVOWARE software https://git.tu-berlin.de/bvt-htbd/facility/tecan_write_gwl/-/blob/master/Tecan_write_gwl/Tecan_write_gwl_for_Raman_measurement.py?ref_type=heads
Set Raman parameters	If you are using the automatic sampling method, the variables for the Raman measurements need to be set in the config file for the run (config_Tecan_write_gwl. json) The parameters used should ideally be the same as the ones used to generate the data with which the model has been trained
Deep clean of the Raman setup	see maintenance
Check liquid levels of canisters	refill system liquid canister with distilled water; empty waste canister

### C.3 Cultivation

In this section the steps necessary to do on the day of the cultivation are detailed. This section is split into three parts: the preparations to do right before the run, what to do during the run and the shut down and cleaning procedure after the run.

#### C.3.1 Preparations Before a Cultivation

In Table C3 we show the steps that need to be performed immediately before the experiment in more detail.

**Table C3:** Preparations right before cultivation.

Step	Details
Start Devices	Switch on the devices: 1. Spectrometer (switches on power supply and back of device) 2. Pump (switch on back of device) 3. Multiplexer valve (switch on side of device) Don't forget to open the shutter of the laser (switch on top of the light guide)
Start servers	In the terminal of the respective project, start the servers for the individual devices, the database and orchestration service. Start the Raman‐orchestrator service last: 1. Raman hive 2. Multiplexer‐valve 3. Ismatec‐pump 4. Spectrometer 5. Raman‐estimate (optional for only measuring spectra) 6. Raman‐orchestator The commands for starting the services and more details can be found in the documentation. Check if errors occur after starting a microservice. If yes, check if the command is correct and if the respective device is switched on properly.
Flush system with water	Flush the system with water to flush out any air bubbles trapped in the system. The command you can use may look like this: *raman‐client queue‐washing‐tasks ‐grpc‐channel 127.0.0.1:50051 ‐clean‐pump‐volume 5e‐3 ‐clean‐flow‐rate 0.75e‐2 ‐pull‐back‐pump‐volume 3.85e‐4 ‐pull‐back‐flow‐rate 5e‐3 ‐number‐of‐wells 8*. Using this command once takes approx. 6 min. More information can be found in the documentation. Flushing at least twice may gives better results.
Check Spectrum Quality	Measure water in the system and compare the spectrum to a good quality water spectrum. Use this notebook for easy evaluation and for more details on the process. If there are any bumps or other irregularities in the spectrum or the spectrum has a low intensity repeat the flushing step or perform a more thorough cleaning (see maintenance). If the spectrum looks good, the system is ready for operation

#### C.3.2 Conducting a Run

Table C4 details the steps necessary to measure Raman spectra during the cultivation run. It distinguishes between 3 modes: *manual*, where the samples are pipetted manually into the interface, *automatic*, where the samples are pipetted by the liquid handling arm and *offline Raman*, where the samples are not measured during the run, but stored until measurement at a later time point.

**Table C4:** During the run.

Step	Details
Sampling	1. Label the plates with: runID<runID>‐<date>‐< operator>‐s< sample‐number>‐raman **Do not use plates with NaOH** (NaOH may form a precipitate with metal ions, interfering with Raman measurement) 2. After samples have been taken: place sample plate into the centrifuge for 5 min, $4^{\circ }$C, max. rpm. 3. After centrifugation, transfer supernatant into last rows off the plate (turning the plate around so that supernatant of container 1 is in upper left corner after flipping the plate) Version 1 (manual): 1. Pipet 50 μL of each sample into the sampling interface using a multichannel pipet 2. Start the measurement by executing the command in the terminal on the computer that runs the orchestrator (*raman‐client queue‐measuring‐tasks ‐grpc‐channel 127.0.0.1:50051 ‐duration 10 ‐num‐scans 2 ‐sample‐volume 50 ‐clean‐pump‐volume 0.00125 ‐clean‐flow‐rate 0.0075 ‐pull‐back‐volume 0.000385 ‐pull‐back‐flow‐rate 0.005 ‐sample‐to‐cuvette‐pump‐volume 0.000115 ‐sample‐to‐cuvette‐flow‐rate 0.0025 ‐move‐sample‐volume 2e‐05 ‐sample‐label <sample‐label> ‐run‐id <run‐id> ‐number‐of‐wells 8 ‐device‐id <device‐id> ‐substrates‐of ‐interest <substrates‐of‐interest>* More information on the command can be found in the documentation 3. Discard the plate after sampling is done Version 2 (automatic): 1. Return the plate to its place on the Tecan deck 2. Initiate measuring by setting isRamanSupernatant_plate_back.gwl to 1 3. The Tecan will measure the samples automatically 4. Discard the plate after sampling is done Version 3 (offline Raman): 1. After all the samplings are taken in the plate, and after centrifugation and transferring of the supernatant, freeze the plate at $-21^{\circ }$C until measurement
Check spectrum quality	Periodically check the quality of the spectra being recorded to make sure that there is no clogging of the pipes or other dirt interfering with the measurements. For more details see part troubleshooting
Regularly check liquid levels of canisters	An empty canister may lead to wrong measurements

#### C.3.3 After a run

In Table C5 the steps that need to be done after the run to shutdown the system are explained in more detail.

**Table C5:** After the run.

Step	Details
Flush the system with water	Flush the system at least twice to remove any residues of samples. The command you can use may look like this: *raman‐client queue‐washing‐tasks ‐grpc‐channel 127.0.0.1:50051 ‐clean‐pump‐volume 5e‐3 ‐clean‐flow‐rate 0.75e‐2 ‐pull‐back‐pump‐volume 3.85e‐4 ‐pull‐back‐flow‐rate 5e‐3 ‐number‐of‐wells 8* (more information in the documentation)
Shut down the servers	Shut down the servers using Ctrl+c
Turn off the devices	Switch off the devices

### C.4 Maintenance

In Table C6 the steps that need to be done periodically to maintain the system and to ensure a high spectrum quality in the long run are shown in more detail. In particular, we perform these steps before a 2mag cultivation.

**Table C6:** Maintenance.

What?	Details	When?
Deep clean	Use cotton swabs and isopropanol or ethanol to clean the pipetting interface, especially inside of the wells: 1. Flush the system with water 2. Use cotton swabs and water to clean the interface 3. Flush thoroughly with water to remove any isopropanol/ethanol traces 4. Check spectrum quality 5. Repeat if necessary	before every run/when needed
Intense clean with acid	Clean the inside of the cuvette and tubes with acid: 1. Disconnect the tube to the system liquid container and insert the tube into acid (5% hydrochloric acid) 2. Flush the system multiple times to remove residues inside the tubes and cuvette 3. Reconnect system liquid and flush thoroughly with water to remove remaining traces of acid	In case of dirtiness, especially inside the tubes or inside the cuvette after working with biological matter

### C.5 Troubleshooting

In Table C7, we outline frequently encountered issues during the measurement process along with their solutions.

**Table C7:** Troubleshooting.

Issue	Possible causes	Possible fixes
The intensity of the measured spectra is very low	There is no or not enough sample	Add sample
	Shutter of Raman probe is closed	Open the shutter
	The cuvette is not positioned correctly	Reposition the cuvette. There should be a spacer in the cuvette holder (with a small hole pointing upwards). If the spacer is in, press down the cuvette onto the spacer. The mirror should be visible through the cuvette.
	There are a lot of cells in the sample	Centrifuge the sample and make sure to separate the supernatant and the pellet properly
	There is another substance besides cells in the sample that increases the optical density of the sample.	Precipitates caused by e.g., NaOH can increase the optical density of the sample, resulting in low spectra intensity. Centrifuge again or try to remove interfering substances from your sample
	There are a lot of air bubbles in the sample being measured	Check the way in which the sample is pipetted in (e.g., pipetting speed of liquid handling station). Check if all connections are tight.
	The cuvette is dirty or clogged	Clean the cuvette intensely with acid (see maintenance)
There is no water coming out of the interface in the flushing step	The system water canister is empty	Refill the canister
	The connections between the tubes are not tight	Retighten all the connections. The connection between the system water canister and the tubes is particularly prone to being untight
The waste‐water in the interface does not flow off	The waste canister is full	Empty the waste canister
	The waste tube has an air lock	Wiggle the tube to release the air or disconnect the tube and connect it again. Check if connections are tight.

## Appendix D List of Material

We provide a list of material used for the microfluidic setup in Table D1.

**Table D1:** List of materials: To connect the devices with each other with tubes, we used the this microfluidic material.

Amount	Article for Setup
10	Nut, flangeless, PPS, $1∕16^{\prime \prime }$ OD, Headless, 1 pc/PAK
2	Flangeless Ferrule Tefzel (ETFE), 1/4‐28 Flat‐Bottom, for $1∕16^{\prime \prime }$ OD blue, 10 pc/PAK
1	Tubing, PTFE, 1∕16×0.02 in ID, 50 ft/PAK
1	Flangeless fitting PEEK 1/16in, 10 pc/PAK
1	Flangeless Ferrule, for 2.0 mm OD, 10 pc/PAK
2	NS1D48042812 ‐ CPC Plug 1/4‐28 UNF Female Thread (Flat Bottom Port), with Shut‐off Valve, EPDM Seal
2	NS1D19042812 ‐ CPC Coupling 1/4‐28 UNF Female Thread (Flat Bottom Port), with Shut‐off Valve, EPDM Seal
1	PMCD4204 ‐ CPC Coupling Plug 6.4 mm hose barb, panel mount, with shut‐off valve, Buna‐N
1	PMCD1704 ‐ Coupling 6.4 mm hose barb, with Shut‐off Valve, Buna‐N Seal
1	Idex ‐ Manufacturer No. P‐387X ‐ Article number GZ‐02022‐09
1	Idex ‐ Manufacturer No. P‐352X ‐ Article number GZ‐02021‐95
2	Huenersdorf^TM^ Wide‐neck canister made of HDPE with UV protection
1	Tefzel (ETFE) Tubing, natural, $1∕16^{\prime \prime }$ OD, $0.020^{\prime \prime }$ ID, 15 m, 1 pc/Pkg
1	Outer nut, flangeless, PEEK, short, headless, M6 Flat‐bottom, for $1∕16^{\prime \prime }$ OD, 10 pc/Pkg
1	Ferrule, flangeless, PEEK, 1/4‐28 flat‐bottom, $1∕16^{\prime \prime }$ OD, natural, 10 pc/Pkg
1	Ferrule, flangeless, with stainless steel ring, 1/4‐28 flat‐bottom, for $1∕16^{\prime \prime }$ OD, natural, 10 pc/Pkg
